# Combined T2 SPAIR, Dynamic Enhancement and DW Imaging Reliably Detect T Staging and Grading of Bladder Cancer With 3.0T MRI

**DOI:** 10.3389/fonc.2020.582532

**Published:** 2020-11-10

**Authors:** Lihua Yuan, Danyan Li, Dan Mu, Xuebin Zhang, Weidong Kong, Le Cheng, Xin Shu, Bing Zhang, Zhishun Wang

**Affiliations:** ^1^ Department of Radiology, Gulou Clinical College of Nanjing Medical University, Nanjing, China; ^2^ Department of Psychiatry, Vagelos College of Physicians and Surgeons, Columbia University, New York, NY, United States

**Keywords:** T2 SPAIR, dynamic contrast-enhanced, diffusion-weighted imaging, bladder cancer, multimodal magnetic resonance imaging

## Abstract

**Objectives:**

To evaluate bladder cancer by integrating multiple imaging features acquired using multimodal 3.0T magnetic resonance imaging (MRI).

**Methods:**

We prospectively enrolled 163 consecutive patients including 142 men (mean age, 65.2 years) and 21 women (mean age, 65.8 years). We evaluated the efficiency and reliability of the multiple imaging modalities including T2-weighted spectral attenuated inversion recovery (SPAIR) imaging, dynamic contrast-enhanced (DCE) imaging and diffusion-weighted (DW) imaging, and the imaging feature, apparent diffusion coefficient (ADC) in the identification of the T staging and grading. We compared our imaging findings with the results of histological examination using McNemar’s test. We reported the results under the significance of p < 0.05. Approval for the study was obtained from the local institutional review board.

**Results:**

The sensitivity and specificity using T2 SPAIR plus DW imaging (sensitivity: 85.2%; specificity: 93.2%), DCE plus DW imaging (sensitivity: 92.4%; specificity: 96.8%), and all the three imaging modalities combined, i.e., T2 SPAIR plus DCE plus DW imaging (sensitivity: 92.5%; specificity: 97.4%), were significantly greater than using T2 SPAIR imaging alone (sensitivity: 74.1%; specificity: 72.2%). One hundred six (93.0%) lesions showed a thin, pedicle arch-like shape and thus primarily demonstrated to be in Ta stage; by contrast, a large number of lesions (137 [85.6%]) were sessile and were found to be in T1 stage. The differences in the ADC were significant between low-grade (877.57 ± 24.15) and high-grade (699.54 ± 23.82) lesions (P < .01).

**Conclusions:**

T2 SPAIR and DCE plus DW imaging provided useful information for evaluating T staging and grading in bladder cancer. Those imaging features to distinguish Ta stage from T1 stage were presented.

## Introduction

Bladder cancer is the most common cancer of the urinary system (1). Globally, it is the ninth most common cause of cancer-related death in humans. The prevalence of bladder cancer in males is three to four times greater than in females. In females, however, bladder cancer is often confirmed with more advanced disease at presentation and less favorable outcomes after treatment (2). Currently, the definitive diagnosis of bladder cancer depends on histological confirmation by cystoscopy or surgery. The contribution of magnetic resonance imaging (MRI) to the diagnosis of bladder cancer has been reported (3). However, conventional images with an echo-planar MR sequence cannot identify primary tumor and can be inaccurate in identifying local staging (4). Based on the opinion of the updated guidelines on bladder cancer, it is necessary to distinguish T1 from Ta cancer then to distinguish the low grade bladder cancer from the high grade one by which it is an important indicator of whether cystectomy is required. MRI with 3.0T may be useful for accurate pretreatment staging, predicting the early response to treatment and providing non-invasive alternatives to cystoscopy for those requiring long-term surveillance, including some advanced scanning modes such as dynamic contrast-enhanced (DCE) and diffusion-weighted imaging (DWI) (5). Because of the fact that different scanning sequences focus on different aspects of imaging features, combination of different sequences can provide more comprehensive and detailed imaging diagnosis of bladder cancer. In addition, based on the opinion of the updated guidelines on bladder cancer (1), more accurate imaging information could be contributed to bladder cancer staging with 3.0T MRI. In this study, we aimed to investigate bladder cancer using the integration of multiple imaging modalities with 3.0T MR; to compare imaging features with pathological results regarding cancer staging; and to evaluate the correlation between apparent diffusion coefficient (ADC) values and histological grade.

## Materials and Methods

### Patients

Between January 2016 and March 2017, 163 patients who presented with gross (macroscopic) hematuria with normal findings from upper urinary tract ultrasonographic evaluation were prospectively enrolled. They were evaluated initially by ultrasonography (US) or cystoscopy. The population included 142 (87.1%) men (65.2 ± 10.6 years old; range, 42–87 years) and 21 (12.9%) women (65.8 ± 10.5 years old; range 57–81 years). For the whole group, the mean age was 65.3 ± 10.2 years old (range, 42–87 years). Single lesions were found in 58 cases (35.6%), and multiple lesions were found in 105 cases (64.4%) (**Table 1**). Patients with cystoscopically proven bladder cancer were subjected to dual-source parallel RF excitation technology MRI and subsequently underwent TUR-BT (transurethral resection of bladder cancer) or radical resection. None of the patients had received TURBT before MRI scanning. Exclusion criteria included upper urinary tract cancer or stones, a history of urinary tract trauma, contraindications to MR imaging (e.g., pacemaker or metallic prostheses) or cystoscopy (e.g., unfit for anesthesia or urethral stricture), and refusal to consent to the study. Approval for the study was obtained from the local institutional review board. Written informed consent was obtained from all of the patients.

**Table 1:** Demographic and Clinical Characteristics.

Characteristic	No. (%) of patients (n = 163)
**Age**	
Age of All Subjects	65.3 ± 10.2 (42–87)
Age of Males	65.2 ± 10.6 (42–87)
Age of Females	65.8 ± 10.5 (57–81)
**Sex**	
Male	142 (87.1%)
Female	21 (12.9%)
**Lesions**	
Single lesions	58 cases (35.6%)
Multiple lesions	105 cases (64.4%)
**Operative technique**	
Transurethral resection of tumor	92 (56%)
Radical/Partial cystectomy	71 (44%)

### MRI Acquisition

Before MRI scanning, proper bladder distension was necessary. Patients were asked to start drinking water half an hour before the MRI and to keep their bladder full at the time of the examination. Checked the bladder filling degree on the image of the localizer, and delayed the examination if the bladder was not full.

All of the measurements on patients were carried out using a 3.0-Tesla imager (Intera Achieva; 3.0T TX, Philips, Best, Netherlands) with respiratory triggering. Axial, orthogonal, high-resolution T2-weighted spectral attenuated inversion recovery (SPAIR), DCE and DW images were acquired sequentially on the same axial orientation using a 16-channel SENSE (Sensitivity-Encoding) abdominal coil. The following parameters were used for T2 SPAIR high-resolution MR of the urinary bladder: TR = 5,000 ms, TE = 110 ms, band width = 50 kHz, 320 × 256 matrix, slice thickness of 3 mm, intersection gap of 1 mm, and field of view (FOV) = 40 cm. DW images were obtained using a single-shot fast spin-echo sequence with chemical shift-selective fat-suppression techniques (b = 0 and 800 s/mm^2^ [DW gradients applied in three orthogonal directions]; matrix, 128 × 128; section thickness, 3 mm; gap, 1 mm; field of view, 30 cm; number of sections, 19–24; number of signals acquired, 14; sensitivity encoding factor, 2; acquisition time, 7 min). Sequentially, T1-weighted DCE imaging was performed to use full time points, including precontrast scanning (with a flip angle of 10°) and dynamic scanning (with a flip angle of 10°) after a single-dose injection of gadopentetate dimeglumine (Omniscan, GE Healthcare, Waukesha, WI, USA) at a dose of 0.1 mmol/kg. The final total scan time was maintained within 4 min. The field of view (AP/RL/FH) was 300/300/200 mm; the voxel size was 8 mm^3^; TR and TE were the limit set by the machine; and the average acquisition times averaged 80 s (range, 0–80 s, 20 time-points and 2 s time interval).

### MR Image Analysis

All MR images were independently examined by two radiologists (DM and XZ, with 10 and 30 years of experience, respectively). The observers knew where the cancer was and ignored all other information. A bi-exponential model was used to describe the behavior of the diffusion-weighted signal in the lesions considered in this study. Seven image sets were reviewed as follows: T2 SPAIR images alone, DCE images alone, DW images alone, T2 SPAIR plus DCE images, T2 SPAIR plus DW images, DCE plus DW images, and all three image types combined. First, the T2 SPAIR alone, DCE alone, DW alone, and T2 SPAIR plus DCE images were interpreted, and then the remaining sets (T2 SPAIR plus DW images, DCE plus DW images, and all three image types combined) were evaluated after 2 weeks. When these three types of images were interpreted together, T2 SPAIR and DCE images were mainly used to identify anatomical structures, and DW images were used to assess the extent of the cancer. The apparent diffusion coefficient (ADC) values of the bladder masses, urine and normal bladder wall were measured.

### Cancer Staging

Differentiation between noninvasive and invasive urothelial cancer is critical to the treatment planning. The non-muscle-invasive urothelial cancer of the bladder (≤T1) requires greatly varying but the unified requirement for risk adaptive treatment and monitoring was to provide thorough care while minimizing the burden associated with treatment. However, the high-stage (≥T2) tumors with high recurrence rate and low progression rate demand intensive care and timely consideration of radical cystectomy (1, 6).

To evaluate the performance and agreement of the two reviewers at identifying bladder tumors the reviewers were requested to classify the cancer into the following two categories (invasive or non-invasive cancer) and subcategories in accordance with the 2009 TNM system of the International Union Against Cancer (7): non-invasive cancer (Tis; Ta; T1) and invasive cancer (T2; T3; T4) (**Table 2**). The staging standard used was similar to the T2 weighted images (9, 10) and contrast-enhanced images (11), and we defined a new standard for DW images, two image types or three image types combined in this study. Because of the difference in the recurrence and progression rates regarding Ta- and T1-stage cancer (6), the cancer size and cancer histological grade were also evaluated.

**Table 2:** T Staging for Bladder Cancer.

Categories	Stage	Description
Non-invasive Bladder Cancer	Tis	Carcinoma *in situ*
	Ta	Papillary non-invasive tumor
	T1	Tumor invades subepithelial connective tissue
Invasive Bladder Cancer	T2a	Tumor invades superficial muscle
	T2b	Tumor invades deep muscle
	T3a	Tumor invades perivesical tissue microscopically
	T3b	Tumor invades perivesical tissue macroscopically
	T4a	Tumor invades prostate, uterus, or vagina
	T4b	Tumor invades pelvic or abdominal wall

Source—Reference (8).

### Definition of Non-Invasive and Invasive Cancer on T2 SPAIR Imaging

**Figure 1:**
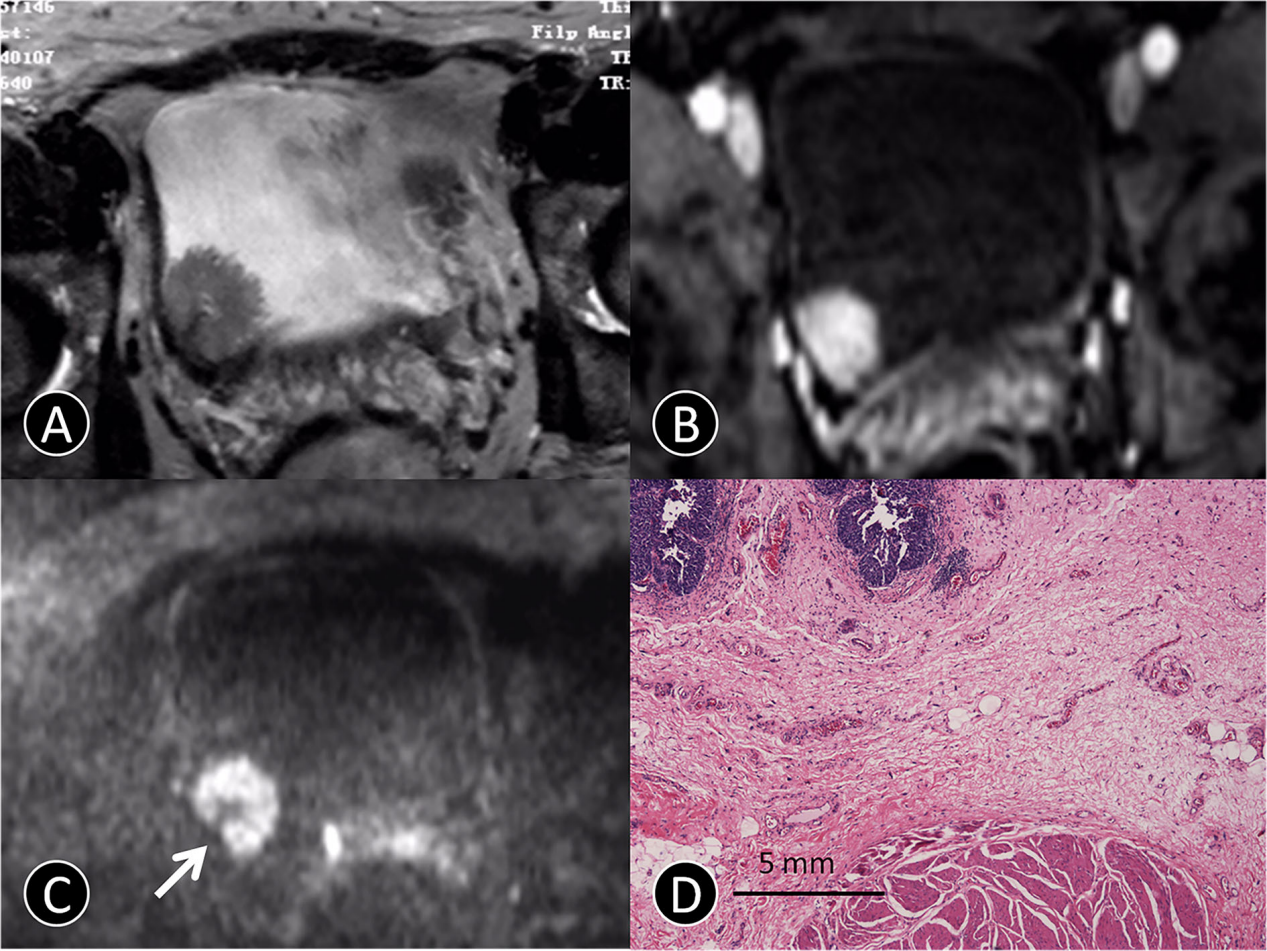
MR images of a 72-year-old man with pTa urothelial carcinoma. **(A)** The transverse T2 SPAIR image shows an oval mass on the right bladder wall without obvious a C-shaped high SI area (arrow). **(B)** The transverse DCE image shows an oval mass that is significantly enhanced, and the submucosa is slightly enhanced without obvious a C-shaped high SI area (arrow). **(C)** The transverse DW MR image shows a C-shaped high SI area with a low SI stalk connecting to the right side of bladder wall with a thin pedicle and small contact area (arrow). **(D)** The photomicrograph of a specimen obtained at TUR shows papillary cancer (blue) with a submucosal stalk (red line) consisting of markedly edematous submucosa, fibrous tissue, capillaries, and mild inflammatory cell infiltration (Hematoxylin-eosin staining; original magnification, ×40).

Since a low signal intensity (SI) line could be observed on the T2 SPAIR image of the normal bladder wall, when the low signal intensity line was obvious (**Figure 1A**), the bladder wall was considered to be intact (≤T1).The bladder was considered to be infiltrated by the cancer (≥T2) (**Figure 3A**) when the low SI line was destroyed focally in the region underlying the cancer (12).

### Definition of Non-Invasive and Invasive Cancer on DCE Imaging

On contrast-enhanced images, submucosal linear enhancement (SLE) is shown immediately after the injection of contrast agent, while SI in muscle layer was still low. Therefore, an integral SLE adjacent to a cancer is indicative of stage Ta (**Figure 1B**). When SLE is disrupted by a cancer, this situation was considered stage T1 or higher disease. On both T2 SPAIR and contrast-enhanced images, cancer extending into an adjacent organ or the abdominal wall was classified as T4 (13).

### Definition of Non-Invasive and Invasive Cancer on DW Imaging

On DW images, bladder cancer shows high SI (14). We assumed that an intermediate SI line delineated the low SI region between the cancer and muscle, which could reflect a muscle layer and a submucosal stalk, respectively (**Figure 1C**). We propose a new DW staging standard: a thin, flat, high SI area corresponding to the cancer or a high SI cancer with a low SI submucosal stalk or a thickened submucosa indicates stage T1 or lower; stage T2 of high SI cancer with smooth margin and no submucous stalk; extension into the perivesical fat with an irregular margin indicates stage T3; and extension into adjacent organs indicates stage T4 (15).

### Histopathologic Analysis

The histopathologic findings of bladder cancer specimens were compared with the preoperative MRI findings for each patient by using the McNemar test. With the difference in cancer biology, growth pattern, and recurrence between low-grade and high-grade bladder cancer, cancer was classified into two grades: low-grade anaplasia and high-grade anaplasia (7).

### ADC Value Measurement

The index of cancer was selected based on the pathological findings in patients with multiple bladder cancers. ADC values, which were used to quantitatively analyze the degree of diffusion for the index cancer, were calculated at a workstation (Philips View Forum R4.1; Philips, Best, Netherlands) using the following formula (16):

(1) $$ADC=\frac{ln(\frac{S(b_{1})}{S(b_{0})})}{b_{1}}$$

where b is the attenuation coefficient (depending only on gradient pulses parameters: (i) gradient intensity and (ii) gradient duration; S(b0) is the MRI signal when b = b0 = 0 s/mm^2^; S(b1) is the MRI signal when b = b1 > 0, where we used b1 = 800 s/mm^2^. A trained image analyst and radiologist manually plotted a contour within a region of interest (ROI) to maximize coverage of index cancer on a transverse ADC map on a slice showing the maximal cancer diameter. The ROI was carefully drawn to exclude the surrounding urine. For cancer with a cancer stalk that showed low signal intensity on DW imaging, the ROI was drawn excluding the stalk. The ADC value of each pixel in the ROI was quantified, and the mean and standard deviation (SD) of the ADC values were calculated. ADC values were measured to estimate the degree of diffusion.

### Statistical Analysis

The data were processed using statistical software (SPSS, version 15; SPSS, Chicago, IL, USA), with conventional cystoscopy or the final histopathologic report as the reference standard. We evaluated the sensitivity, specificity, accuracy, positive predictive value(PPV), negative predictive value (NPV), and Cohen’s kappa coefficient (κ) (to measure inter-rater reliability) of T2 SPAIR, DCE and DW images to identify bladder cancer and the cause of the hematuria. A comparison of imaging findings with cystoscopy and histology was subsequently performed using the McNemar test. The ADC values of histological low-grade and high-grade urothelial cancer were compared using t test. A p value less than 0.05 was considered to indicate statistical significance.

## Results

### Optimization of Imaging Protocols

The sensitivity, specificity, and overall accuracy of the consensus of the two observers for differentiating cancer (≤T1) from cancer (≥T2) are summarized in **Table 3**. The sensitivities and specificities using T2 SPAIR plus DW imaging (sensitivity: 85.2%; specificity: 93.2%), DCE plus DW imaging (sensitivity: 92.4%; specificity: 96.8%), and all the three imaging modalities combined, i.e., T2 SPAIR plus DCE plus DW imaging (sensitivity: 92.5%; specificity: 97.4%), were significantly greater than using T2 SPAIR imaging alone (sensitivity: 74.1%; specificity: 72.2%). The accuracies achieved using T2 SPAIR plus DW images (90.1%), DCE plus DW images (93.6%), or all the three image types combined (95.2%) were also greater than the accuracy achieved using T2 SPAIR images alone (73.0%).

**Table 3:** Diagnostic Accuracy for Differentiating Cancer Stage (≤T1) from Cancer stage (≥T2).

Imaging Set	Sensitivity	Specificity	Accuracy	PPV	NPV	κ Value	P Value
T2 SPAIR	74.1%	72.2%	73.0%	0.50	0.92	0.70	<0.05
DCE	80.2%	85.7%	84.3%	0.56	0.90	0.88	<0.05
DW	83.7%	90.3%	90.0%	0.67	0.93	0.63	<0.05
T2 SPAIR +DCE	84.1%	86.3%	83.5%	0.71	0.91	0.55	<0.05
T2 SPAIR +DW	85.2%	93.2%	90.1%	0.54	0.86	0.76	<0.05
DCE+DW	92.4%	96.8%	93.6%	0.67	0.93	0.88	<0.05
T2 SPAIR +DCE+DW	92.5%	97.4%	95.2%	0.80	0.93	0.91	<0.01

PPV, positive predictive value; NPV, negative predictive value.

Interobserver agreement of each interpretation is summarized in **Table 3**. Interobserver agreement of all the three imaging modalities (T2 SPAIR plus DCE plus DW imaging) combined was the highest (κ = 0.91, p < 0.01) compared to T2 SPAIR plus DW images (κ = 0.76, p < 0.05) and DCE plus DW images (κ = 0.88, p < 0.01).

### Cancer Characteristics

The 163 patients with 375 tumors were used to evaluate the ability to differentiate T1 and lower cancers from T2 and higher cancers. The pathologic stage was between Ta and T1 in 73% (274 of 375) of tumors, and T2 or higher stage cancers occurred in 27.0% (101 of 375) of tumors. T2 or higher stage tumors were divided into T2 (17% [64 of 375]), T3 (6% [22 of 375]), and T4 (4% [15 of 375]) regarding pathology. The cancers measured 0.61–88.5 mm in maximum diameter (mean, 24.6 mm). Histological diagnoses were all urothelial carcinoma (n = 363) and urothelial carcinoma with adenocarcinoma (n = 12). The histological grade was low grade in 120 (31.9%) of the 98 tumors and high grade in 255 (68.1%) of the tumors.

### Differentiation of Ta Cancer From T1 Cancer

**Figure 2:**
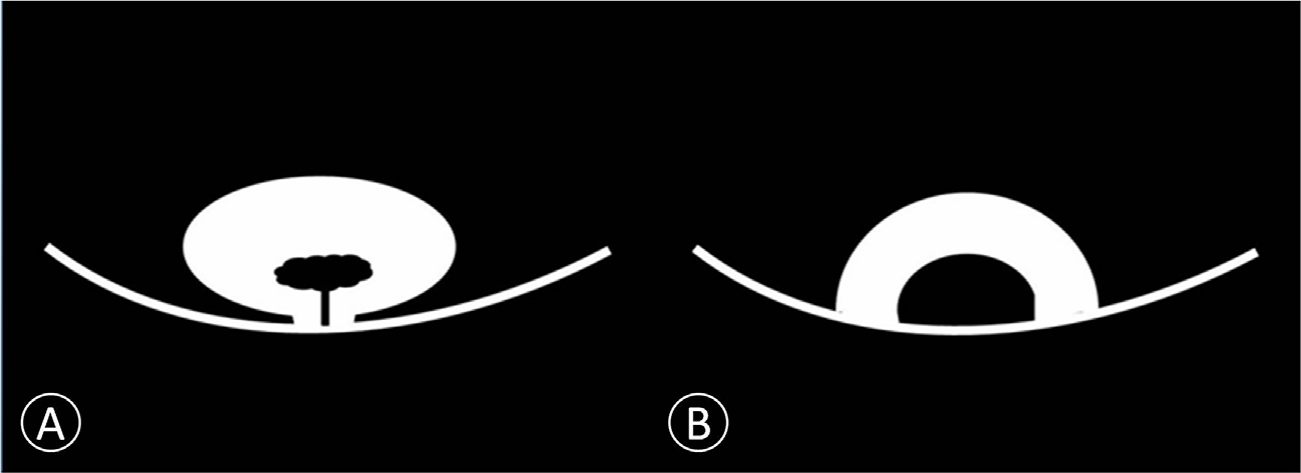
Schematic diagram of non-invasive bladder cancer. **(A)** Cancer is connected to the bladder wall by a thin pedicle with a small contact area. We proposed this model as papillary cancer that could be observed on DWI. **(B)** Cancer is connected to the bladder wall with a wide base contact area. We proposed this model as sessile cancer that could be observed on DWI.

Specific features of non-invasive cancer (Ta and T1) are summarized in **Table 4**. Because of the difference in cancer biology, growth pattern, and recurrence between low-grade bladder cancer with T1 stage and high-grade bladder cancer with T1 stage, it is necessary to distinguish T1 from Ta cancer before to distinguish low-grade bladder cancer from high-grade. It is not necessary to distinguish Ta with low-grade from Ta with high-grade bladder cancer. No significant difference was found in the number and size of cancer between the Ta and T1 groups in each patient (**Table 4**). However, papillary cancer accounted for most of stage Ta bladder cancer, and the main feature of T1 stage cancer was an arch-like shaped sessile tumor with a wide base (**Figure 2** and **Table 4**). Most of the Ta cancers were low grade, whereas most of the T1 cancers were high grade (**Table 4**).

**Table 4:** Related Indicators in Non-invasive Bladder Cancer (Ta and T1).

		Ta	T1	P Value
No. of tumors in each patient		2.1	1.9	0.12
Cancer size		35 mm	37 mm	0.09
No. of arch-like shapes on DWI	Papillary	106	8	<0.05
	Sessile	23	137	<0.05
No. of tumors according to histological grade	Low-grade	62	31	<0.05
	High-grade	73	108	<0.05

### Comparison of Imaging Findings and Histopathology as the Gold Standard

**Figure 3:**
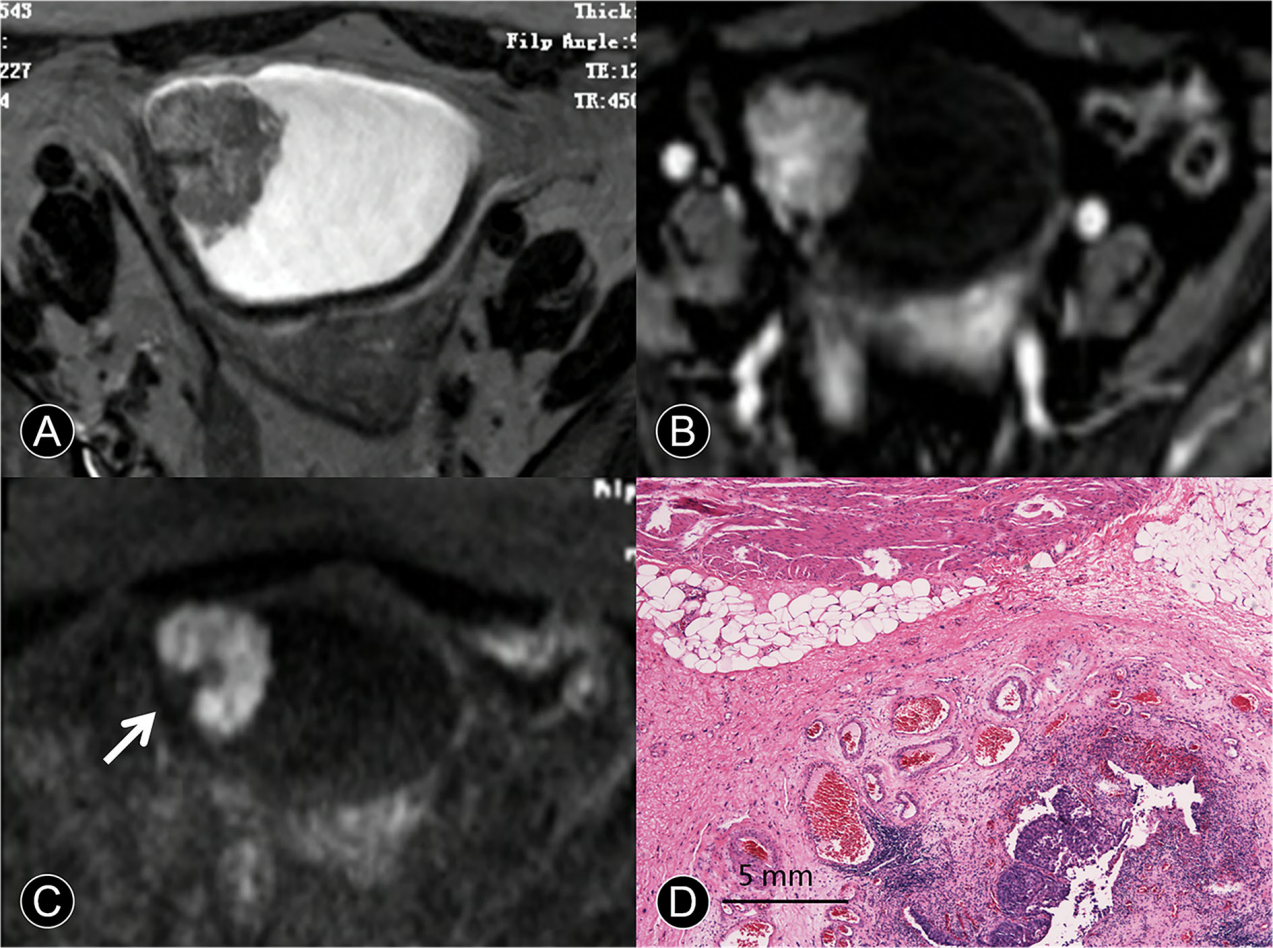
MR images of a 65-year-old man with pT1 urothelial carcinoma. **(A)** The transverse T2 SPAIR image shows an oval mass on the right discontinuous bladder wall without obvious a C-shaped high SI area (arrow). **(B)** The transverse DCE image shows an oval mass that is enhanced, the central part of the mass is significantly enhanced, and the submucosa is slightly enhanced without obvious a C-shaped high SI area (arrow). **(C)** The transverse DW MR image shows a C-shaped high SI area with a low SI stalk connecting to the right side of the bladder wall with a wide base contact area (arrow). **(D)** The photomicrograph of a specimen shows papillary cancer invading the submucosa (Hematoxylin-eosin staining; original magnification, ×40).

**Figure 4:**
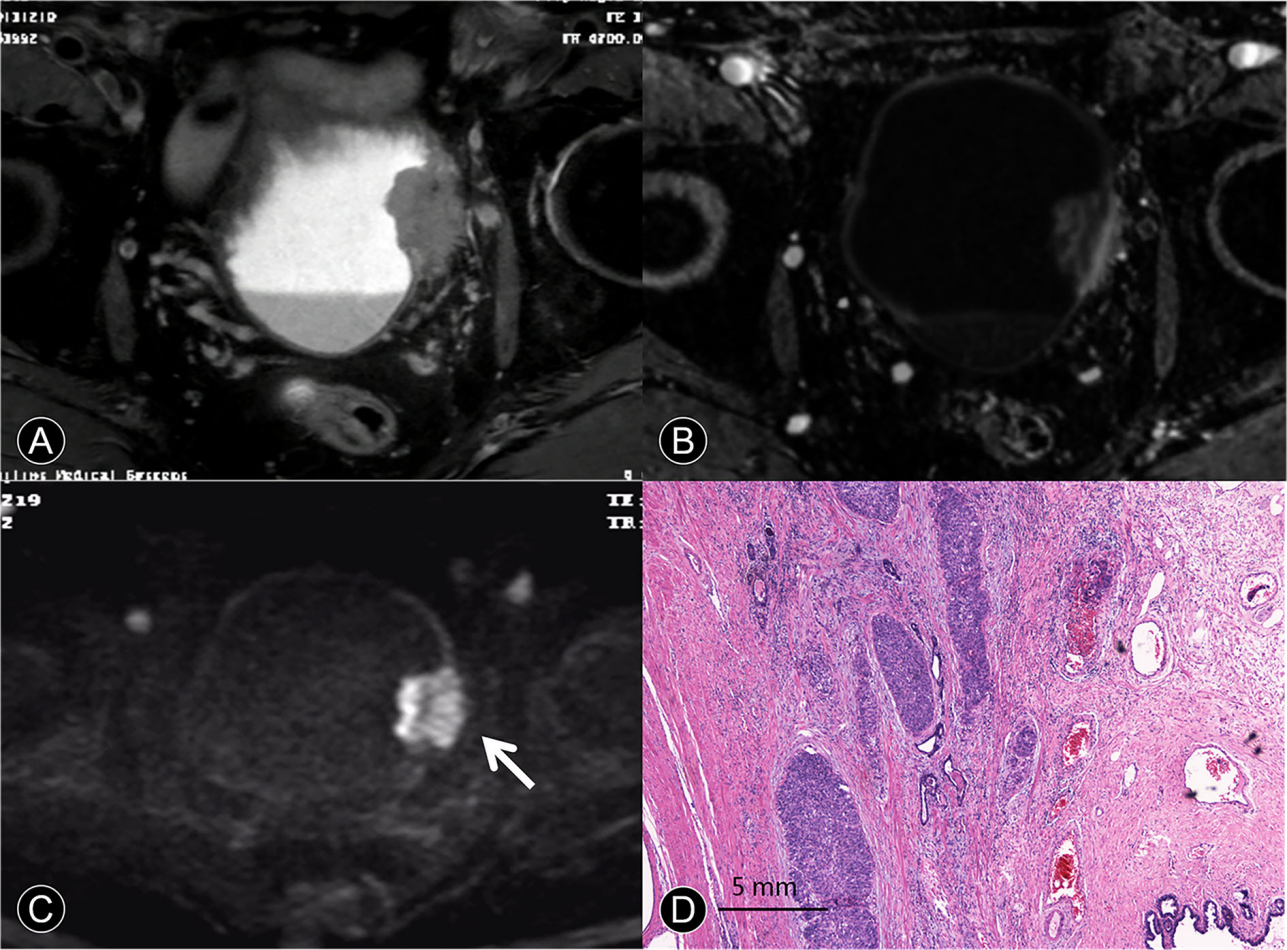
MR images of a 63-year-old man with pT4a urothelial carcinoma. **(A)** The transverse T2 SPAIR image shows large nonpapillary cancer on the deformed muscle layer. The SI of the muscle layer at base of the cancer is elevated, and there is clear evidence of perivesical invasion. **(B)** The DCE image of the axial section leftward to the wall of the bladder does not depict cancer contour because microvessels surrounding the cancer are also enhanced. **(C)** The transverse DW image shows a large cancer with an irregular margin spreading toward the surrounding fat tissue (arrow). **(D)** A photomicrograph of the specimen shows papillary cancer invading the muscular layer and prostate (Hematoxylin-eosin staining; original magnification, ×40).

Image quality, despite some distortions commonly observed on DW-MRI, was sufficient in all 163 patients to allow for interpretation. The three hundred seventy-five tumors obtained using radical cystectomy (n = 85) or TUR (n = 290) were available for histopathologic correlation. The phase of enhancement of the submucosal tissue was earlier than that of the cancer component in Ta cancer at dynamic phases (98/129 [76%]; **Figure 1B**). However, the cancer component enhanced as strongly as the submucosal tissue at all dynamic phases in T1 cancer (131/145 [90.3%]; **Figure 3B**). In terms of histopathology, the high, intermediate, and low SI areas on DW images corresponded well to cancer, smooth muscle, and submucosal connective tissue, respectively (**Figures 1A, D**). The DW imaging finding of high SI bladder cancer together with a low SI submucosal stalk resembled an arch-like inchworm shape and was found in 268/274 (97.8%) patients with ≤T1 disease (**Figure 3C**). All of the invasive urothelial cancers showed a smooth or slightly irregular contour or irregular margins toward the perivesical fat, a finding that correlated with pathologic findings (**Figure 4**).

### ADC and Histological Grade

**Figure 5:**
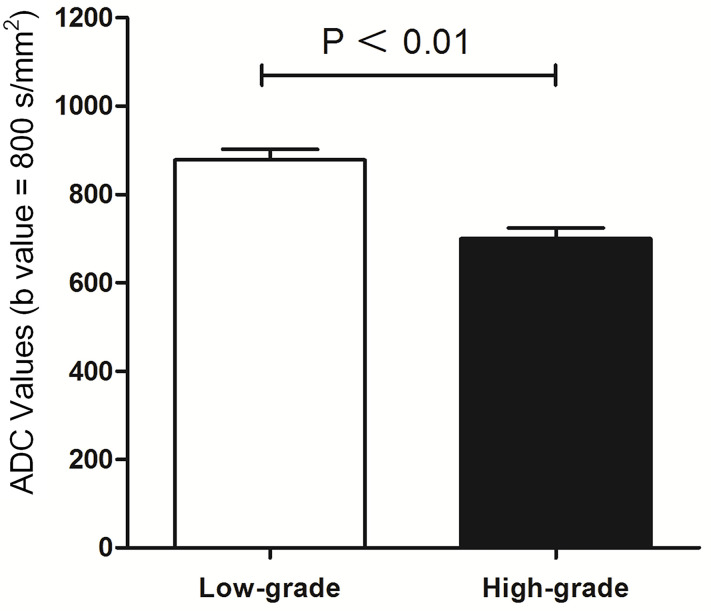
Comparison between histological low-grade and high-grade urothelial cancer according to ADC value (mm²/s).

The correlation between ADC and histological grade is summarized in **Figure 5**. The mean ADC of the 375 bladder tumors was 788.56 ± 21.27 × 10^-3^ mm^2^/s. The differences in ADC values were significant between low-grade with 877.57 ± 24.15 × 10^-3^ mm^2^/s and high-grade tumors with 699.54 ± 23.82 × 10^-3^ mm^2^/s (P < 0.01).

## Discussion

We have systematically evaluated multiple MRI modalities including T2 SPAIR, DCE, and DW imaging and their combination in the diagnosis of bladder cancer, specifically in staging and grading of cancers at stages ≤T1 (tumor invades subepithelial connective tissue) and at stages ≥T2 (tumor invades superficial and deep muscles), which is of great significance in clinical applications. We have found that the integration of T2 SPAIR imaging, DCE imaging, and DW imaging yields the highest sensitivity, specificity, accuracy, observer-agreement among single one or any two combinations of them. We also found that the apparent diffusion coefficient (ADC) data extracted from DW images are significantly different between low- and high-grade cancers that are determined by using histological data, indicating that the ADC data have a unique potential in objectively identifying the grade of bladder cancer without using invasive histological diagnosis.

The preoperative evaluation of different stages of bladder cancer with MRI is a viable and secure tool for surgical patients. The accuracy of the distinction between stage T1 or lower and stage T2 or higher has been reported to be approximately 75% to 95% (8, 17). The overall accuracy for diagnosing cancer stage is approximately 52% to 93% (8, 17, 18). Hayashi et al. showed the accuracy of 52–93%, and the overall diagnostic accuracy of 83% using an endorectal coil (8). Takeuchi et al. showed the accuracy of 92%, and the overall staging accuracy of 98% using a cardiac coil (17). In our study, the accuracy was 95% when a body coil was used (**Table 3**). This value was higher than that in Hayashi’s report (8) but slightly lower than Takeuchi’s report (17). The causes might be the different employment of the coils and different size of samples. Additional causes could be related to the different sizes of the field of view (FOV) or other differences in the scanning parameters. It was found out that DCE images combined with DWI could contribute to improving the accuracy significantly. A possible cause is that the DCE images might show the cancer margin, component, bladder muscle layer and submucosa in different forms of enhancement throughout the different phases. The DCE images acting in a complementary way and a potential benefit of the combined use of DWI that detected the phenomenon of water molecule movement were further explored. It was reported that DW images are useful not only in cancer staging but also in being a reference model for other sequences (19). Bladder is a hollow muscular structure that pumps fluids using peristaltic motion. MRI is not good enough at staging each detail of the hollow organ. For example, it is not easy to distinguish T2 from T3, as well as T3a from T3b, well and accurately. Additionally, with the thin bladder wall and even thinner muscular structure, the low resolution of DWI is not good at observing the cancer margin and normal muscular structure. Fortunately, according to histology, genetics research and new guidelines (1, 6, 7, 20), radical cystectomy was suggested to treat patients with higher stages of bladder cancer from T2 to T4a. It was reported that T2-weighted imaging was not sufficient to distinguish cancer from the muscle layer of bladder. Cancer and the muscle layer have similar SI in 81% of T2-weighted images (18). However, T2 SPAIR techniques provided improved the insensitivity to field heterogeneity (21). The diagnostic evaluation of combined T2 SPAIR, DWI and DCE images in bladder cancer T stage would be advocated for better assay results (**Table 3**).

The judgment of the cancer margin or depth invaded in the bladder wall is helpful for the recruitment and selection for surgery (22). The width of the inflammation zone and width of the submucosa were detected between normal tissues and cancer of the bladder (**Figure 1D**). These facts suggest that the border between normal tissues and cancer of the bladder could be determined by the structural difference between the tissue components using MRI. The high-quality images of MRI could help to detect the localization of the bladder cancer cell boundary (9).

The typical crab-like appearance of cancer was not noted in bladder cancer from Ta to T1 stage. The results showed that the total detection of cancer with integration of multiple imaging modalities was better than any imaging alone (P < 0.05; **Table 3**). The possible reasons for the difference might be that the basic principle of T2 SPAIR and dynamic contrast-enhanced MRI provides evidence of anatomy and contrast enhancement. DWI is extremely sensitive to any net translational movement of water molecules, and its signal intensity is influenced by many factors (23). Therefore, the integration of all of the modalities is better than any used alone.

MR Imaging features of bladder cancer are essential for cancer staging (18, 24). Saito et al. (25) reported that the stalk extending from the bladder wall to the center of the cancer consisted of capillaries, inflammatory cells, fibrous tissues, and edema. Our results showed that pathological tissue related to the enhancing region in the early phase of dynamic contrast agent-enhanced images and the low SI area found on DW images in the center of the cancer primarily consisted of edematous submucosa, fibrous tissue, capillaries, and mild inflammatory cell infiltration (**Figures 1D**, **2D**, and **3D**). These features were frequent on dynamic contrast agent-enhanced and DW images for all T1 or lower cancers confirmed by pathology, findings that were similar to those in a previous report (17). The imaging feature of the stalk might correspond to the low-stage bladder cancer with microvessels and reactive tissue by long-term inflammatory. Additionally, the difference in imaging stage from Ta to T1 would be related to the size of the tumor in contact with the bladder wall (**Figure 2** and **Table 4**).

In the treatment of localized, invasive bladder cancer, the standard treatment remains radical surgical removal of the bladder within standard limits (1). For patients with inoperable locally advanced tumors (T4b), primary radical cystectomy is a palliative option and is not recommended as a curative treatment (1). Therefore, it is important to perform a preliminary assessment of cancer boundaries and determine whether the pelvic or abdominal wall is invaded on MRI. In our study, the sensitivity, specificity, and accuracy achieved using T2 SPAIR, DCE plus DW imaging for diagnosing T4 tumors were all high and are summarized in **Table 3**. DCE plus DW images appear to provide useful information for evaluating cancer contours and size. To our knowledge, no detailed reports have been published concerning T4 bladder cancer with DCE plus DW images. Thus, it would be beneficial to comprehensively evaluate the scope of T4 invasion.

The choice of surgical approach is based not only on the clinical staging of bladder cancer but also on the combination of a comprehensive cancer grade assessment (1, 6). Bladder cancer grades are based on the blood supply and morphologic features of cancer cells as reported previously (26). ADCs representing the degree of restriction of water molecules or diffusivity are inversely correlated with the tissue cellularity and integrity of the cell membranes (27). ADCs have been successfully applied to other parts of the human body in cancer grading (28, 29). Matsuki et al. reported that ADCs of bladder cancer were lower than those of the surrounding structures (14). In our study, the mean ADC of high-grade cancer was significantly lower than that of low-grade cancer (P < 0.05), and all high-grade cancer had an ADC less than 699.54 ± 23.82 mm^2^/s (b = 800). There was a high correlation between ADCs and histological grade (Z > 0.8) that was different from that in previous reports, in which cancer was divided into G1 to G3. Our research combined ADCs with the latest bladder cancer grading. Although the evaluation of cancer grading from ADCs is influenced by several factors (29), the ADCs might still partly predict the histological grade of bladder cancer.

Our study had a number of limitations. First, tumors were divided into noninvasive and invasive urothelial tumors from T1 to T4. There were no accurate methods of differentiating sub-staging, for example, as well as no emphasis on Tis and discrimination between T3a and T3b. These differences in classification might have led to a higher accuracy. The division method is closely related to the development level of MRI and surgical treatment. In addition, the distribution of T stage was uneven with a large number of T1 or lower cancers and a small number of pT2 or higher cancers. The cause may be that patients with stage T1 or lower cancers constitute the main segment of the bladder cancer population. Additionally, our primary objectives were to discriminate between stage T1 or lower cancer and stage T2 or greater cancer, a factor that was crucial for the appropriate treatment of patients with bladder cancer.

In conclusion, the method of T2 SPAIR, DCE plus DW images provided useful information for the more accurate evaluation of T stage in bladder cancer, particularly for differentiating Ta from T1 or lower cancer from T2 or higher cancer. The 3.0T MR imaging features between Ta and T_1_ of bladder cancer were presented. The combination of T2 SPAIR, DCE plus DW images plays a crucial role in the staging and grading of bladder cancer.

## Data Availability Statement

The raw data supporting the conclusions of this article will be made available by the authors, without undue reservation.

## Ethics Statement

The studies involving human participants were reviewed and approved by the Ethics Committee of Nanjing Drum Tower Hospital. Written informed consent was obtained from all of the patients.

## Author Contributions

LY designed the study and contributed to its conception. LY, DL and ZW were major contributors in the writing of the manuscript. Acquisition of data was by DM. Analysis and interpretation of data were by XZ and WK. LC and XS checked the experimental data and provided advice. BZ and ZW revised the manuscript for important intellectual content. All authors contributed to the article and approved the submitted version.

## Funding

This work was supported by the Project of Nanjing health and Family Planning Commission (YKK17089).

## Conflict of Interest

The authors declare that the research was conducted in the absence of any commercial or financial relationships that could be construed as a potential conflict of interest.
